# Bioinspired Diatomite Membrane with Selective Superwettability for Oil/Water Separation

**DOI:** 10.1038/s41598-017-01642-2

**Published:** 2017-05-03

**Authors:** Yu-Hsiang Lo, Ching-Yu Yang, Haw-Kai Chang, Wei-Chen Hung, Po-Yu Chen

**Affiliations:** Department of Materials Science and Engineering, National Tsing Hua University, Hsinchu 101, Sec. 2, Kuang-Fu Rd., Hsinchu, 30013 Taiwan

## Abstract

Membranes with selective superwettability for oil/water separation have received significant attention during the past decades. Hierarchical structures and surface roughness are believed to improve the oil repellency and the stability of Cassie-Baxter state. Diatoms, unicellular photosynthetic algae, possess sophisticated skeletal shells (called frustules) which are made of hydrated silica. Motivated by the hierarchical micro- and nanoscale features of diatom, we fabricate a hierarchical diatomite membrane which consists of aligned micro-sized channels by the freeze casting process. The fine nano-porous structures of frustules are well preserved after the post sintering process. The bioinspired diatomite membrane performs both underwater superoleophobicity and superhydrophobicity under various oils. Additionally, we demonstrate the highly efficient oil/water separation capabililty of the membranes in various harsh environments. The water flux can be further adjusted by tuning the cooling rates. The eco-friendly and robust bioinspired membranes produced by the simple, cost-effective freeze casting method can be potentially applied for large scale and efficient oil/water separation.

## Introduction

Noticing that the serious issue of worldwide oil spill nowadays, more and more researchers seek alternative or improved methods to purify oily-contaminated water. Various substrates including commercially available meshes^1–4^, filter papers^5^, membranes^6–8^, and foams^9–11^ possess different affinities to oil/water systems mainly by superhydrophilic/oelophobic^3, 12^ and superhydrophilic/underwater superoleophobic surfaces^6, 13, 14^ that show great potential to extend the application in oil/water separation. Underwater superoleophobic materials have been extensively studied by several groups recently^15–18^. They obtain hierarchically structured surface and good water adsorption capability to keep the water near the surface to minimize the contact area between oily liquids and solid surfaces^15^. Distinguished from the conventional superoleophobic materials in air, those surfaces are capable of repelling oil or organic solvents only when immersed into water or being wet. They provide a new design principle of antifouling surfaces for the applications, such as separation, transportation, and collection in aquatic/marine environments. However, one major challenge of these artificial textured surfaces lies in the durability and the superwettability performance when the surfaces subjected to external mechanical impact.

Biomimetic and bioinspired materials are emerging fields for learning the strategies from nature and leading to sustainable solutions. Several natural surfaces have been demonstrated to be underwater superoleophobic, such as shark denticles^19^, fish scales^20^ and the abaxial surface of lotus leaves^21^. Those surfaces possess resistant to oily contamination^3^ because of the selective superwettability underwater. Diatoms, the unicellular, photosynthetic microalgae, ubiquitously appear in aqueous environments and play a crucial role for the eco-system by providing ~25% net primary production of oxygen on earth. There are more than 100,000 classified diatom species^22^. The unique and intricate morphologies of diatoms have fascinated scientists and engineers in different fields in the past decades^23–25^. The skeleton of diatom, called frustule, is composed mainly of amorphous silica which displays a diversity of structures and morphologies at the nano-, submicro- to micrometer-scales^26^. Diatoms demonstrate, in an elegant way, how organisms build the sophisticated structures from the bottom-up approach by simple elements. The diatomaceous earth (or diatomite) is the fossilized remain of diatom which exhibits high surface/volume ratio, water absorbing/retention, eco-friendly and chemically inert^27^. Inspired by the unique properties of diatomite, we report bio-inspired diatomite membranes with hierarchical structures that exhibit robust underwater superwettability and superior functional tolerance to a broad range of physical and chemical challenges for the first time. By applying the freeze casting technique^28–32^, an ice-templating process, the bioinspired diatomite membrane with highly anisotropic micro-channels and well preserved nano-porous structures can be fabricated. The synthesized diatomite membrane exhibits both superhydrophilicity and superoleophilicity in air. Surprisingly, the membrane exhibits not only superoleophobicity underwater, but also hydrophobicity under oil. Such dual lyophobic surface in oil-water system is scarcely reported and discussed^33^. In addition, we manipulate the freezing front velocity to adjust the porosity and formation of lamellar structure of the green body. Superior oil/water separation efficiency and satisfactory chemical stability are demonstrated by the bioinspired diatomite membrane. The long-term durability and reliability, which has seldom been performed, are also investigated and discussed in this study.

## Results

### Bioinspired diatomite membrane

**Figure 1:**
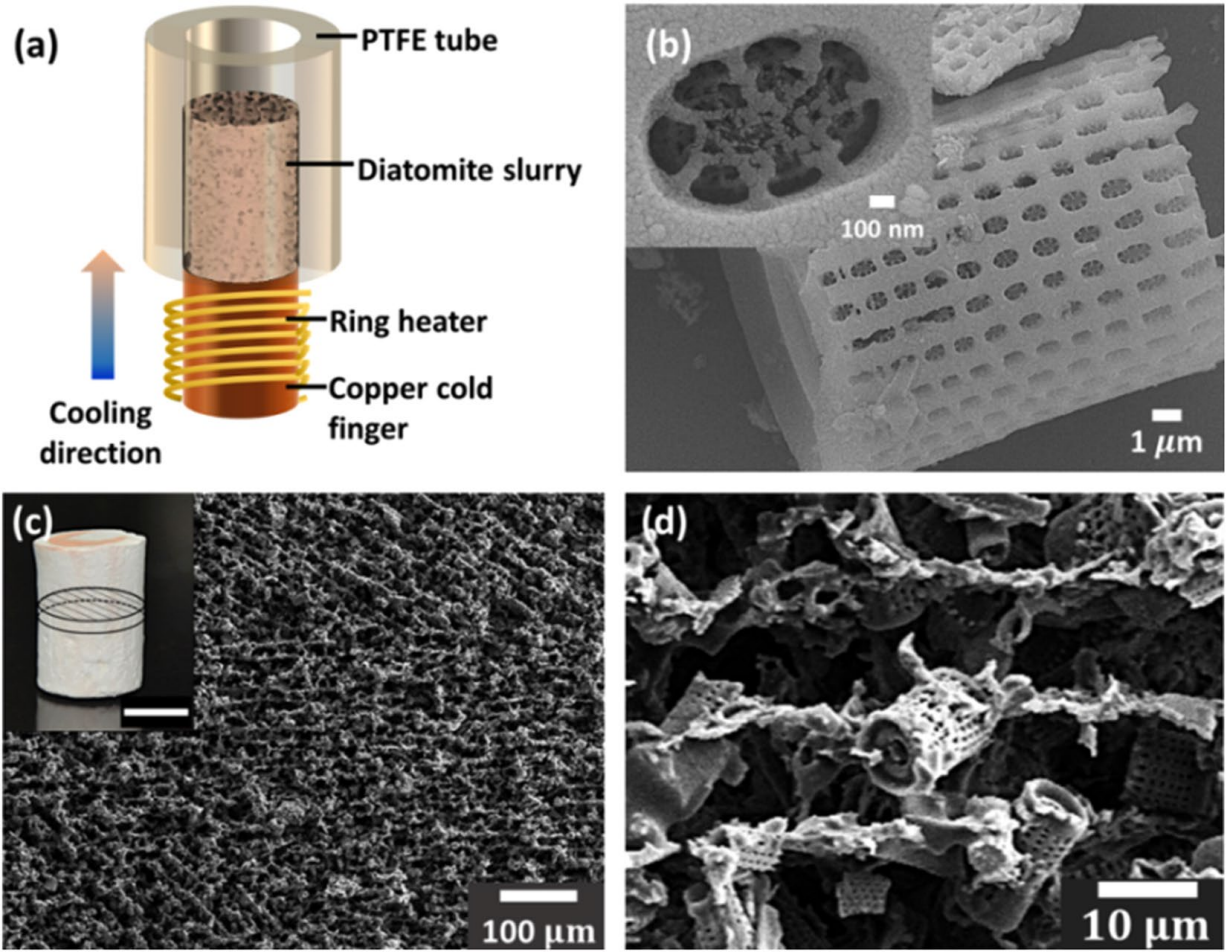
(**a**) Schematic illustration of the freeze-casting system. FE-SEM images show (**b**) the hierarchical structure of a single diatom frustule (Inset: the nano-pores on the silica frustule at high magnification), (**c**) cross-sectional view of the diatomite scaffold synthesized at cooling rate of 5 °C min^−1^, and (**d**) detailed microstructure of diatomites at higher magnification. Inset in (**c**) is a photograph of as-synthesized diatomite scaffold (scale bar: 1 cm).

Figure 1a illustrates the working principle of freeze casting process for synthesizing the hierarchically structured diatomite membrane. The purchased diatomite is composed of faultless diatomite and broken frustules. The micro- and nano-scale structural features of diatom frustule are shown in Fig. 1b. Most complete frustules are cylindrical in shape, 5–15 μm in diameter and 10–15 μm in height. The nano-porosities embedded in the silica wall are of hundreds of nanometers in diameter. The water-based diatomite slurry with organic binder and dispersant was poured into the Polytetrafluoroethylene (PTFE) tube. Under a steady unidirectional cooling flux provided by the copper cold finger, the ice crystals solidified and formed lamellar structures by expelling diatomites within the slurry. The frozen diatomite sample was further put into a chamber under reduced pressure and increasing temperature to sublimate the ice crystals without altering the synthesized lamellar structures. After sintering, the bioinspired diatomite scaffold with highly anisotropic microstructures can be obtained.

To have a comprehensive understanding of the porous structure for diatomite scaffold, we first characterize the morphology of an as-synthesized diatomite scaffold by field emission scanning electron microscopy (FE-SEM), as shown in Figure S1. We clearly observed three distinctive zones, namely dense, cellular, and lamellar zones, from the longitudinal section of an as-synthesized scaffold in Figure S1a. At the very beginning of the solidification (40 μm from the bottom part), the diatomite is assembled into a compact structure. No obvious micro-scaled channels or pores are formed, and this is the region referred as the dense zone (Figure S1d). In the second zone (40–300 μm), the material displays a cellular morphology. Several pores with size ranging from 5 to 13 μm are found on the structure (Figure S1c). From 300 to 3500 μm, there is a transition region where the structure transforms from cellular to lamellar structure. The structure firstly forms lamellae with tilted orientation, then the orientation changes gradually and finally parallel to the cooling direction. In the upper zone (>3500 μm), the structure is lamellar, with elongated and interconnected channels aligned parallel to each other densely along the cooling direction, and exhibits a more organized and uni-directional morphology throughout this region (Figure S1b). The upper zone (lamellar structure) is considered suitable for filtration due to the uni-directional parallel pore configuration, which might decrease the path length for water transportation and increase the water flux. Moreover, for scaffolds synthesized with cooling rate 5° C/min, the lamellar structure accounts for around 86% of the whole scaffold, which gives enough volume for subsequent oil-water separation. In this research, the as-synthesized scaffold was subsequently sectioned into membranes 2 mm in thickness by a rotating diamond blade for the following tests.

The morphology of the transverse section for diatomite membrane was as shown in Fig. 1c. The lamellae structures are arranged in an interwoven manner and with elongated channels between adjacent lamellae. The dimensions of the synthesized channels were identified from the morphology under higher magnification, as shown in Fig. 1d. The membranes cut from the scaffold with cooling rate of 5 °C min^−1^ contain channels sized 15 ± 0.2 μm in short axis and 30 ± 10 μm in long axis and the average lamellae thickness is 8.4 ± 0.3 μm. Characterized by a white light confocal microscope, the root-mean-square roughness (R_q_) of diatomite membrane is measured as 25.7 μm ± 2.7 μm, and the mean value of surface roughness (S_q_) is measured as 29.5 μm ± 2.8 μm from the three dimensional topography image as shown in Figure S2a and b. To intuitively determine the surface chemistry of the diatomite membrane, we employed X-ray photoelectron spectroscopy (XPS) technique. As shown in Figure S3a, the full XPS spectra indicated that silicon, oxygen, sodium, and aluminium mainly exist on the surface of diatomite membrane. As shown in Figure S3b, the characteristic peak at 102.7 eV corresponds to aluminosilicate. The chemical shift with respect to Si2p peak can be attributed the difference in the Si-bonding environment and binding energy of the resulting silicon-containing compound is dependent on the number of Si-O bonds per Si atom. Additionally, we also observed Al2p peak at 74.1 eV which is coincident with the result from Si2p peak that there are some of aluminosilicate embedded into the frustules (Figure S3c). To identify the phase of diatomite, we utilized X-ray diffractometer (XRD) to characterize diatomite powder before and after sintering, as shown in Figure S4. Before sintering, the raw diatomite is identified as amorphous silica phase. Whereas after sintering, the diatomite shows increasing peak intensities with the characteristic peaks corresponding to crystalline cristobalite phase with tetragonal structure (space group *P*41212).

### Selective wettability in liquid systems

**Figure 2:**
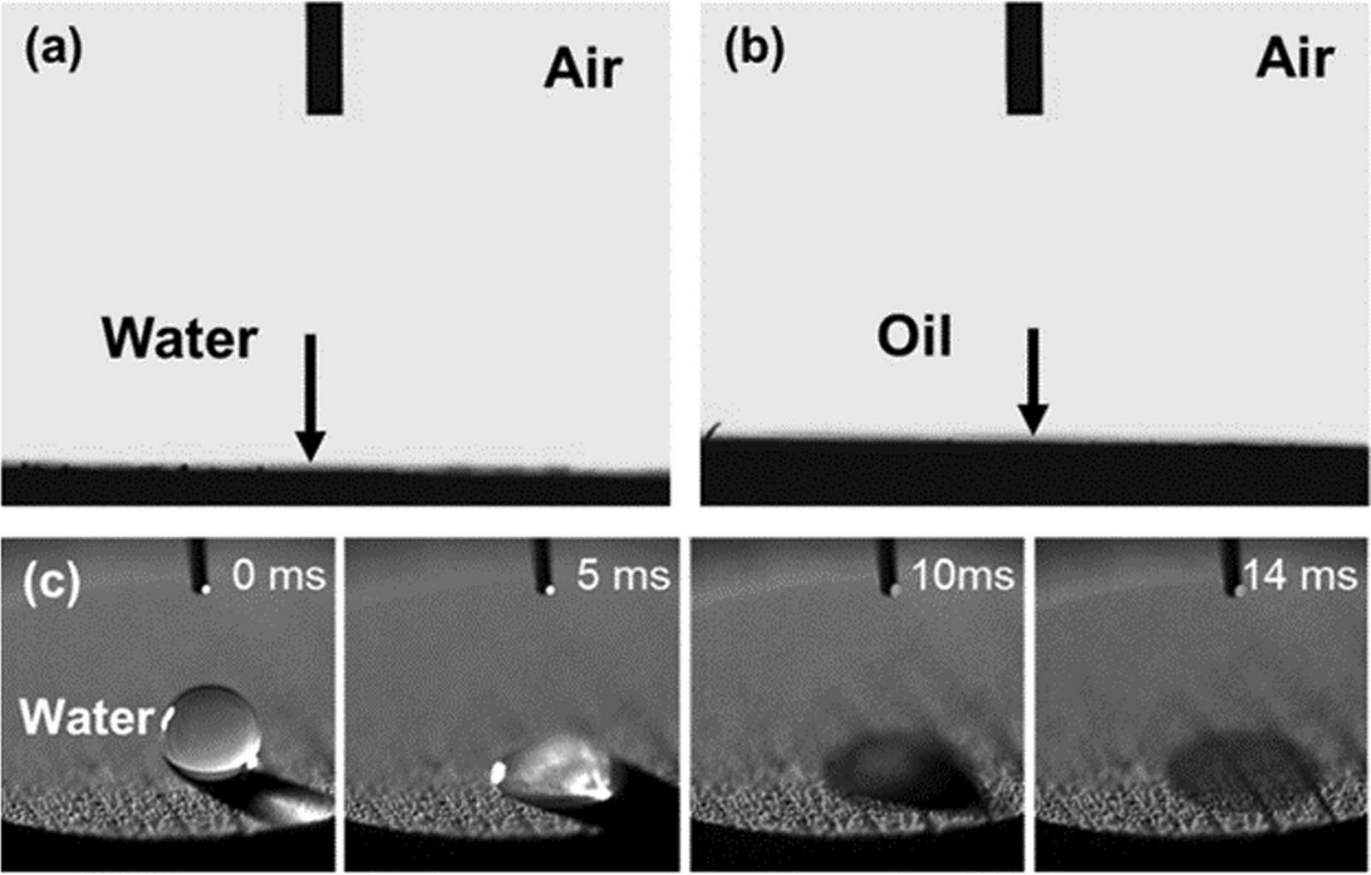
The contact angles of (**a**) water and (**b**) oil on the hierarchical porous membrane (cooling rate: 5 °C min^−1^). (**c**) Still images captured from a video showing a water droplet penetrates into the membrane within 14 ms.

As shown in Fig. 2a and b, the bioinspired diatomite membrane exhibits both superhydrophilicity and superoleophilicity in air. Water and oil droplets penetrate into the membrane immediately, and the static contact angles of water and soybean oil (**γ**_***OA***_ = 30.5 ± 1.0 mN m^−1^, 25 °C) are nearly 0°. We further characterized the wetting time of water by a high speed camera, as shown in Fig. 2c. The still images from video reveal that a water droplet with 7.7 µL penetrates into the diatomite membrane within less than 14 ms, which is much faster than the reported works^2, 34^.

The diatomite membrane is mainly composed of silica and aluminosilica, which are intrinsically hydrophilic. In addition, owing to the hierarchical pores of frustules and micro-size channels fabricated by freeze casting, it is energetically favourable only if the pores are either filled or empty^35^. Therefore, the synergistic effect of surface chemistry and porous structures renders the surface of diatomite membrane superamphiphilic in air. On the other hand, we investigated the wetting properties of diatomite membrane in oil-water systems. To avoid the effect of humidity adsorption on diatomite membrane, the membrane was firstly heated under approximately 100 °C in a vacuum 24 hours, and subsequently immersed into the tested oil for another 24 hours right after being taken out from the oven. We discovered that if the membrane containing trace amount of water molecules, the contact angle toward water under oils will become lower and the water droplet could either adhere to the surface or even penetrate through the membrane. Once the membrane is dry enough by heat treatment, the porous diatomite membrane becomes a liquid-infused slippery surface when immersed into oils. The effect of humidity is relatively minor for the contact angles of oil in water.

**Figure 3:**
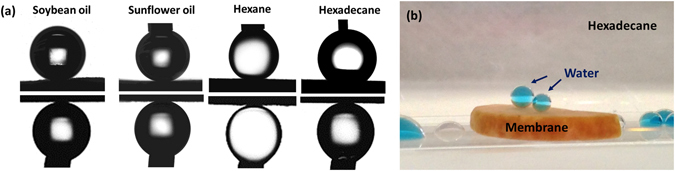
(**a**) Droplet profiles for wetting properties of the diatomite membrane in oil-water systems. Upper row: water droplets in soybean oil, sunflower oil, hexane and hexadecane, respectively. Lower row: the droplets of the aforementioned oils in water. (**b**) Photograph of a prewetted membrane by hexadecane. The two blue-dyed water droplets showed high contact angles on the membrane. The droplets surrounded by the membrane are those rolling off the surface of membrane.

In Fig. 3a, the diatomite membrane showed the dual superlyophobicity in oil/water systems. All water droplets, including cooking oils (e.g. soybean oil, sunflower oil) and non-polar oils (e.g. hexane, hexadecane) showed high contact angles (>150°) and low roll-off angles (<10°) on the oil-prewetted and water prewetted diatomite membranes, respectively (Tables S1 and S2 summarizes the contact angles in oil/water system for tested liquids). When the diatomite membrane was immersed in water, the underwater contact angles against both soybean oil and hexadecane exceed 165°. Distinct from the wettability measured in air, the diatomite membrane in water shows superior selective superwettability against oils and various organic alkanes. The underwater contact angles of diatomite membrane with cooling rate of 5 °C/min are 169.4° ± 1.3° for soybean oil, 167.1° ± 0.4° for hexane, 167.0° ± 0.9° for hexadecane, 168.3° ± 0.7° for dodecane, 170.2° ± 1.7° for light crude oil, and 171.1° ± 1.3° for heavy crude oil. Heavy crude oil is defined by low American Petroleum Institute (API) gravity (<20° API), i.e., high density and viscosity (>10^3^ cP, 25 °C) due to heavier molecular compositions^36^. Light crude oil is composed of lighter molecular compositions, resulting in lower density and viscosity. The results indicate that the diatomite membrane shows superior superoleophobicity underwater for both non-polar and polar oil, even for viscous liquids.

According to the snapshots shown in Figure S5b and c, we discovered that the adhesion between the oil or water droplets and the membrane in oil/water systems is extremely low. Even after squeezing the liquid droplet on the surface for approximately 5 seconds, no obvious shape distortion of oil droplet was observed when oil left the surface. Furthermore, we did not observe the residue of either oil or water left on the surface after droplet retraction. We attribute the underwater superoleophobicity and low oil adhesion of the diatomite membrane to the immiscibility between water and organic oils. When the membrane was immersed into water, based on Young’s equation (*γ*_*OA*_ × cos*θ*_*OA*_ + *γ*_*SO*_ = *γ*_*WA*_ × cos*θ*_*WA*_ + *γ*_*SW*_), the value of interfacial energy of oil/silica (*γ*_*OS*_) can be calculated larger than that of water/silica (*γ*_*SW*_), because the contact angle of water (*θ*_*Wa*_ = 34.9° ± 1.7°) is larger than that of soybean oil (*θ*_*OA*_ = 26.3° ± 1.1°) on a flat amorphous silica slide in air. Therefore, a continuous water layer formed within the porous structures of diatomite membrane immediately. The wetted diatomite membrane, which is energetically favourable to be underwater superoleophobic, can be considered as a liquid-infused surface. Since oils and water are intrinsically immiscible, the water layer near the surface acts as a buffer layer that minimizes the contact area between oil and substrates and prevents the absorption of oil. The oil droplet tends to form the spherical shape to minimize surface energy, thus resulting in high underwater contact angle. Therefore, the oil adhesion is significantly decreased.

The underwater contact angle of oil droplet *θ*_*OW*_ on an water-infused surface, which can be considered as a smooth surface, is expressed by^37^:

1 $$\cos \,{\theta }_{OW}=\frac{{\gamma }_{SW}-{\gamma }_{SO}}{{\gamma }_{OW}}=\frac{{\gamma }_{OA}{\rm{\cos }}{\theta }_{O}-{\gamma }_{WA}{\rm{\cos }}{\theta }_{W}}{{\gamma }_{OW}}$$

where *γ*_*OW*_, *γ*_*SW*_, *γ*_*SO*_, *γ*_*OA*_, *γ*_*WA*_ are surface tensions of the oil-water, solid-water, solid-oil, oil-air, and water-air interfaces, respectively. *θ*_*O*_ and *θ*_*W*_ represent contact angles of oil and water measured in air. Based on eq (1), increasing the hydrophilicity of one solid surface leads to an increase in the oleophobic property in water because *γ*_*OA*_ is usually much lower than *γ*_*WA*_.

Schematic in Figure S5a illustrates the mechanism of the diatomite membrane with selective under liquid superwettability. According to thermodynamics, an underwater superoleophobic surface is not energetically favorable, in general, for exhibiting good water repellency under oil. Surfaces with higher surface energy tend to obtain stronger affinity toward water than oil; yet low surface-energy surfaces show stronger affinity to oil. Surprisingly, the bioinspired diatomite membrane can achieve such high contact angle against water when immersed into oil systems. Based on our knowledge, only a handful of papers studied such surfaces with superlyophobicity in both oil and water systems^33^. We believe that the dual superlyophobicity of diatomite membrane is induced by superamphiphilicity in air, because the hierarchical porous structures can trap liquid molecules within the structures tightly and repel other liquids which are immiscible in the infused liquid of membrane. It is different from the aspect discussed in Tian’s work^33^ which showed the structured surfaces with intermediate wettability (56° < θ_sca_ < 74°) guaranteeing Cassie states for both water-in-oil and oil-in-water cases if the surface condition can satisfy both criteria regarding water/oil filling and outward suspension force provided by the geometry.

Based on these results, the oil-fouling problem is less likely to occur on the diatomite membrane. Due to such high underwater contact angles and low adhesion against oil droplets, the bioinspired diatomite membrane is a good candidate for the application of oil-water separation.

### Chemically Stability and Separation Efficiency

**Figure 4:**
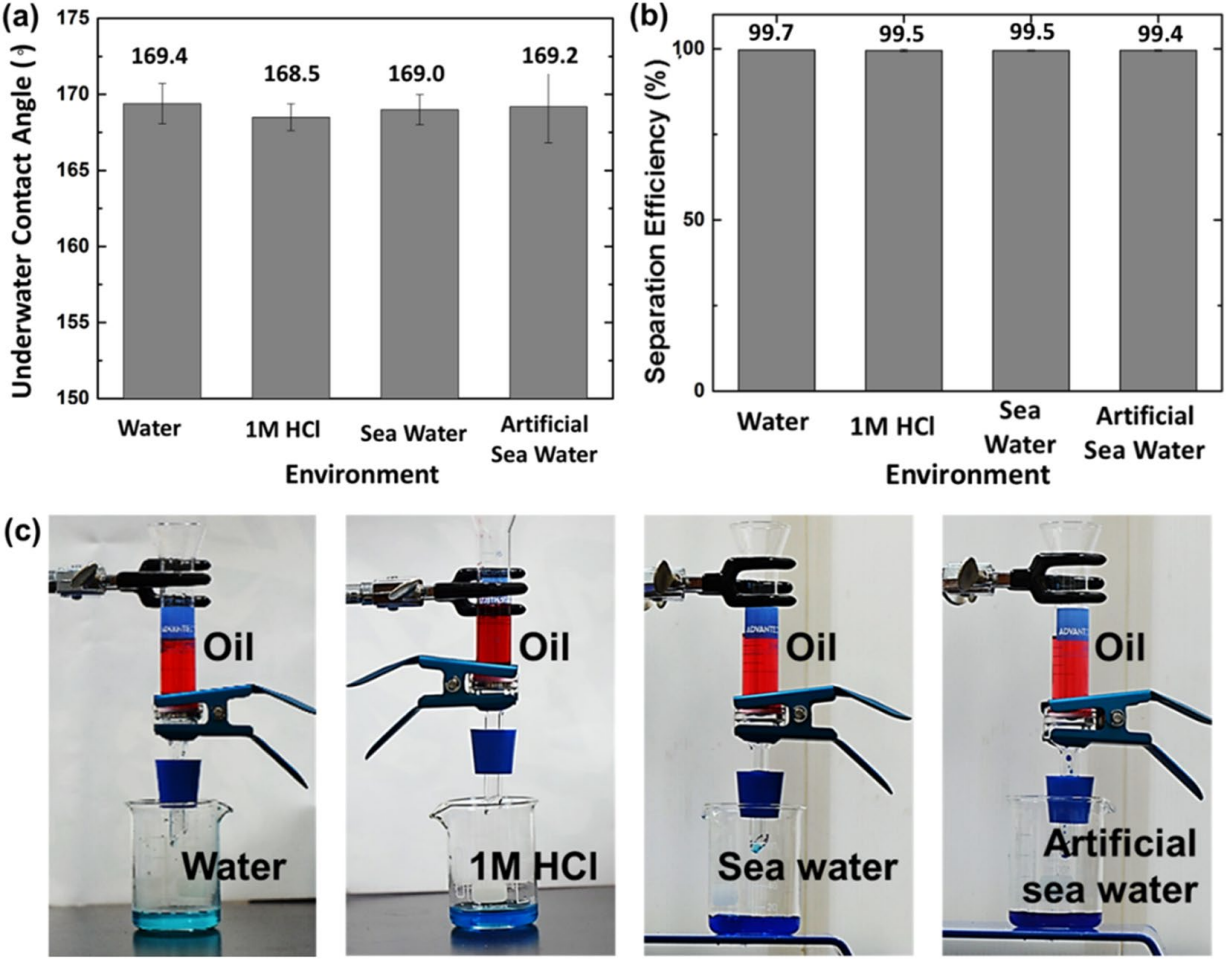
(**a**) The oil contact angle tested under different environments. The plates (cooling rate: 5 °C min^−1^) show superoleophobicity in pure water, 1 M HCl solution, sea water and artificial sea water. (**b**) The free oil/water separation efficiencies tested under different environments (V_oil_ = 10 mL, V_liquid_ = 10 mL) and (**c**) the photograph of the separation process.

To demonstrate the oil repellency under harsh environments, we utilized a membrane with cooling rate of 5 °C min^−1^ for further testing. The underwater contact angles were measured after immersing in 1 M HCl solution, sea water, and artificial sea water for 24 hours. As shown in Fig. 4a, no decrement of the value of underwater contact angle in various environments was observed. All the samples showed remarkable stability for underwater selective wettability against oil even after long-time immersion in aforementioned liquids. We demonstrated that a bioinspired diatomite membrane possesses stable chemical inertness and robustness under severe environments. It shows great potential for the continuous separation of oily wastewater produced by industries or the oil spill accidents in the ocean.

An as-synthesized diatomite membrane was subsequently used for oil/water separations after sectioned into 2 mm thick. The hierarchically-structured diatomite membrane was fixed between two glass tubes. The membrane was pre-wetted by water and immersed into water to make sure all the entrapped air cushions were removed. 20 mL of free oil/water mixture (V_water_/V_oil_ = 1:1) without surfactant was poured into the upper tube. The images of experimental setup and procedure of the separation process are as shown in Figure S6a and b. Due to the superhydrophilicity and underwater superoleophobicity of the pre-wetted membrane, it only allows the water to pass through quickly but the oil is retained on the top tube, showing satisfactory separation performance. The separation efficiency is calculated by the following equation^38^.

2 $$E( \% )=\frac{{m}_{i(water)}}{{m}_{0(water)}}\times 100 \% $$

Here, *m*_*i*(*wate*)_ is the weight of water after separation, and *m*_0(*wate*)_ is the weight of water before mixing with oil prior to the experiment. The hierarchical porous diatomite membrane not only separates free oil/water mixture with high efficiency (99.7%), but also shows high performance under acidic and briny environments, both reach over 99.5% (Fig. 4b and c). The free soybean oil/water mixture prepared before the filtration and the liquid collected after the filtration are shown in Figure S7a and b. The filtrate remained clean and transparent without visible oil residue under an optical microscope observation, even after 30-cycle filtration.

### Intrusion Pressure Measurement

The intrusion pressure measurement is shown in Figure S6c. The diatomite membrane can sustain over 80 cm of soybean oil, and there is no visible oil breaking through during the experiment or even after one-hour idling. The bearing pressure can then be calculated by ***P*** = ***ρgh***, which reaches ≈7.2 kPa (density of soybean oil ≈915 kg/m^3^)^39^. The intrusion pressure is defined as the maximum pressure the material can sustain, and the oil will start to flow downward and penetrate the material when exceeding this critical pressure. Since 80-cm height is the upper limit of our device used in this experiment, we expect that the bioinspired diatomite membrane can sustain even higher intrusion pressure than 7.2 kPa. The theoretical intrusion pressure can be calculated by the Young-Laplace equation^13, 17^:

3 $${P}_{theoretical}=-\frac{2{\gamma }_{OW}\cos {\theta }_{OW}}{r}$$

where *γ*_*OW*_ stands for the oil/water interfacial tension, *cosθ*_*OW*_ is the oil contact angle underwater, and *r* is the pore radius. Eq (3) is the balance between the external force and the capillary force. For *P*_*theoretical*_ > 0, the oil contact angle underwater *θ*_*OW*_ must be larger than 90°, or the external force will be at the same direction with the capillary force, and the oil flows down immediately. The intrusion pressure is also directly proportional to the reciprocal of the pore radius (*r*).

By inserting *γ*_*OW*_ ≈ 30 mN m^−1^, *θ*_*OW*_ = 169.5°, *r* ≈ 11.1 μm (calculated by the average of the short and long axis of the channel), the theoretical intrusion pressure can be obtained, which gives *P*_*theoretical*_ ≈ 5.3 kPa. It can be clearly observed that the experimentally measured intrusion pressure is higher than the theoretical value. This may due to the theoretical intrusion pressure only considers the surface with identical pore size while the intrusion situation in the diatomite membrane is far more complicated. The as-synthesized plate has a complex three-dimensional architecture possessing not only micro-sized channels but also submicro/nano-scaled roughness and pores provided by frustules, which can trap a large amount of water within these structures and forms a more stable water/oil interface. As a result, the experimentally measured intrusion pressure is higher than the theoretically predicted value. The diatomite membrane can sustain at least 7.2 kPa of oil, indicating promising operation potential for the separation of free oil/water mixture.

### Durability and anti-fouling property

**Figure 5:**
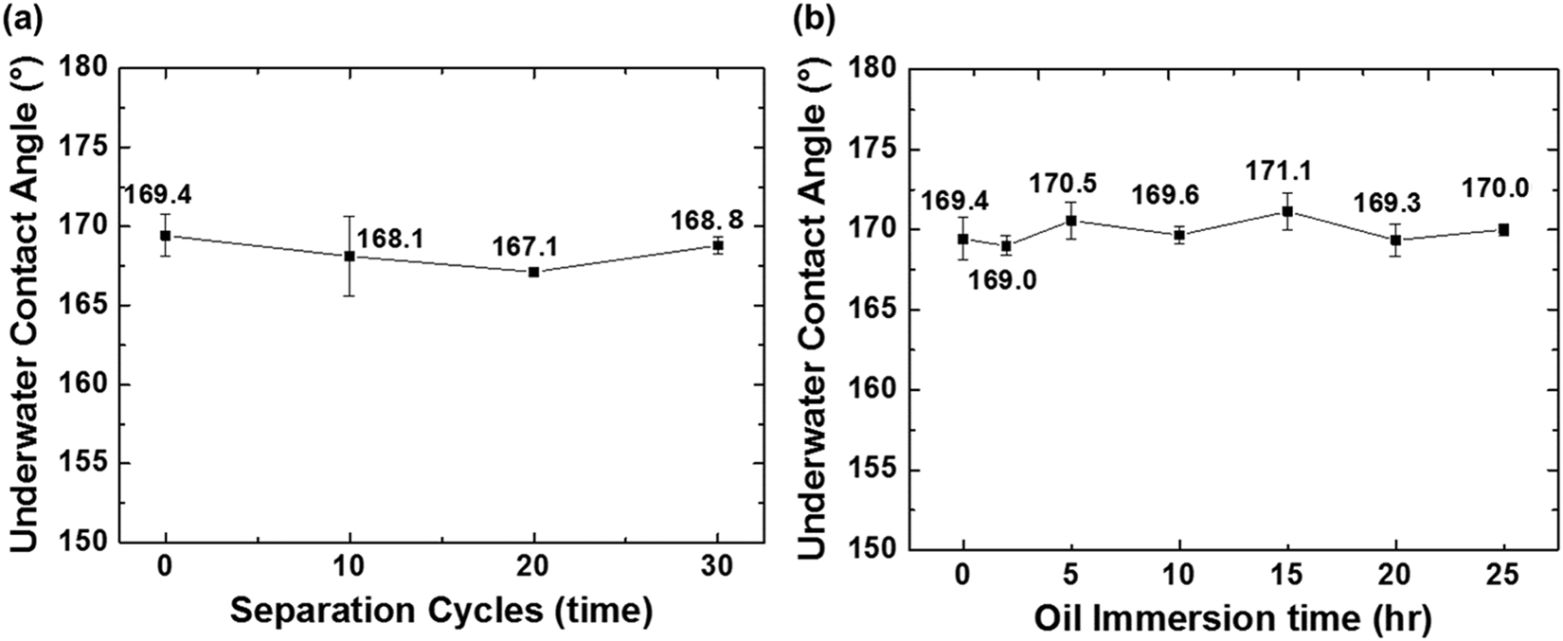
Underwater contact angle measurements on diatomite membrane synthesized with cooling rate of 5 °C min^−1^ (**a**) after different separation cycles, and (**b**) after immersing in a soybean oil bath for a period of time.

To further investigate the durability of the diatomite membrane for oil/water separation, we applied the membrane for 30 separation cycles. As shown in Fig. 5a, the surface maintains stably high underwater contact angle toward oil. The filtrate was observed under an optical microscope, and no visible oil residue was found. We also evaluated the long-term oil repellency of diatomite membrane by directly immersing the pre-wetted diatomite membrane into soybean oil bath. After certain period of time, the immersed membrane was taken out from the oil bath and flushed by deionized water before measuring the underwater contact angle toward oil. In Fig. 5b, long-term immersion into oil bath did not compromise the capability of oil repellency underwater. Two photographs of diatomite membranes taken during the oil bath immersion reveal an obvious water plastron (bright, reflective layer) surrounding on the pre-wetted diatomite membrane (Figure S8b), yet not on the one without pre-wetting (Figure S8a). Thus, the pre-wetted membrane obtains better antifouling capability against oil. The observation concurs with our hypothesis that how the sample traps water within the hierarchical structure, and reduces the contact area with oil. Since the sample only allows the water to pass through and the stable oil/water interface formed at the surface, it can be used for a large number of separation cycles without oil-fouling problem and has great potential for long-term applications.

**Figure 6:**
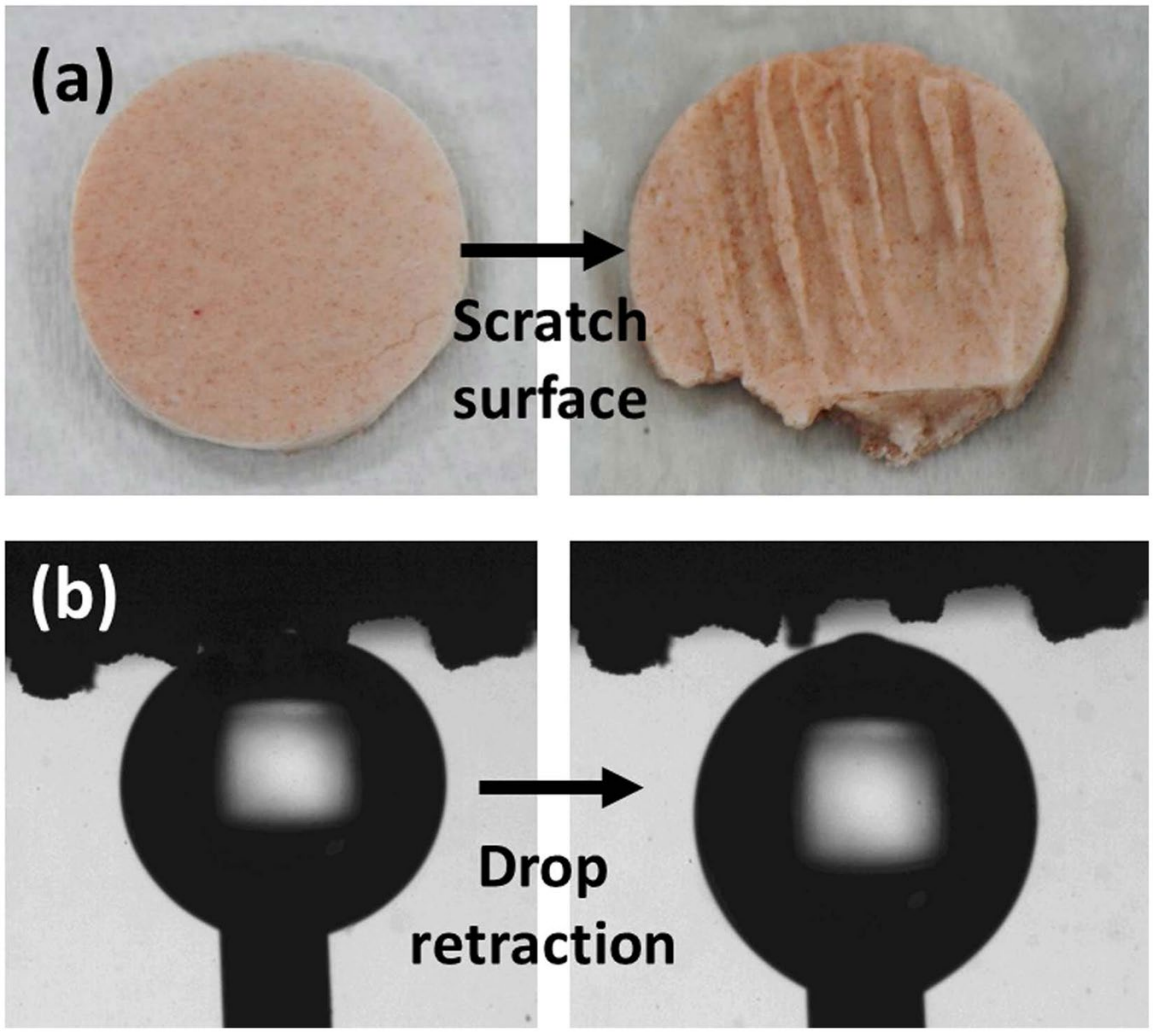
Evaluation of mechanical stability. (**a**) Photographs of scratched diatomite membrane. (**b**) The oil droplet profiles of the scratched membrane for demonstrating low adhesion between oil droplet and surface.

We further evaluated the mechanical durability of the membrane by making several scratches on the surface of diatomite membrane with a fine tip stainless steel tweezer. As shown in Fig. 6a, several deep and long scratches were made on the pre-wetted diatomite membrane. When immersed in the water, those scratches on the membrane can be clearly seen by CCD camera. The bioinspired membrane still exhibited superior underwater superoleophobicity after suffered the severe mechanical damage on the surface. High underwater contact angle and extremely low adhesion between oil droplet and the scratched surface were demonstrated in Fig. 6b.

### Water Flux Measurement

**Figure 7:**
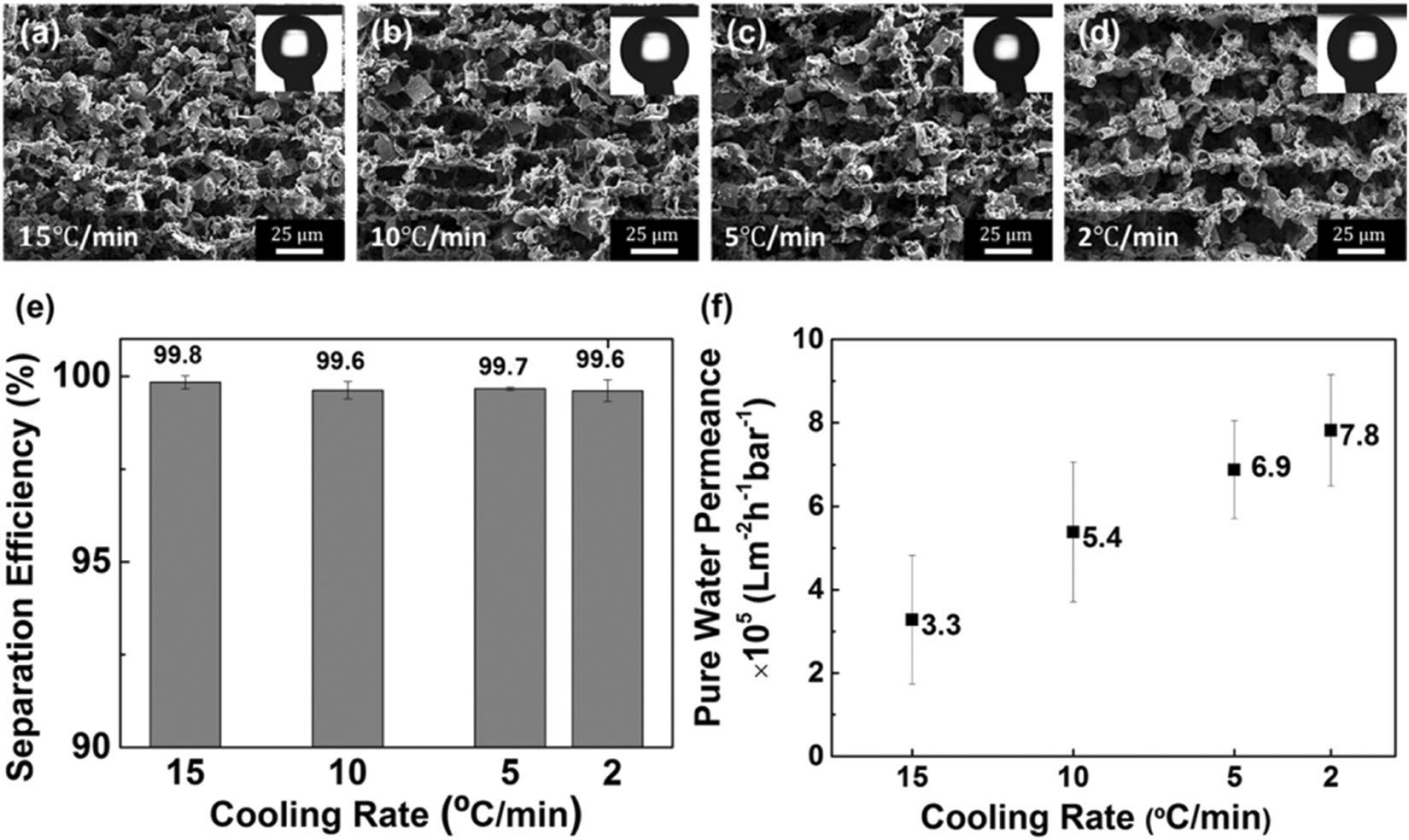
(**a**–**d**) Influence of the cooling rates on the microstructures of diatomite membranes. (**e**) Separation efficiency and (**f**) pure water permeance tested by plates synthesized with different rates.

The structure of the diatomite membrane can be tuned by controlling the cooling rate during the freeze casting process. Figure 7a to 7d show the transverse section of the plates synthesized at cooling rates of 2, 5, 10, and 15 °C min^−1^. The short axis of the pore and lamellae thickness decrease significantly with increasing cooling rate, from an average channel width of 13.3 μm (2 °C min^−1^) to 8.8 μm (15 °C min^−1^), as shown in Table S3. The underlying reason is attributed to the different growth velocities of the ice crystal structure, where the growth rate along the cooling direction is much faster than that of perpendicular to the cooling direction^40^. When the cooling rate is increased, there is not enough time for the ice crystal to grow along the transverse direction, thus creating finer microstructure with reduced pore size and wall thickness.

Samples synthesized with cooling rates of 2, 5, 10, and 15 °C min^−1^ also show superoleophobicity underwater, and the measured separation efficiencies are reported in Fig. 7e. All samples freeze casted at different cooling rates can reach over 99.6% efficiency, and there is no obvious difference in the separation efficiency among samples. The filtrate was further observed under an optical microscope, and no trace of oil can be found.

The separation efficiency does not change significantly with the diatomite membranes freeze casted under different cooling rates. Pure water flux, however, is directly affected by the cooling rate of the plate (channel size). For samples prepared with cooling rates of 2, 5, 10, and 15 °C min^−1^, the corresponding pure water fluxes are 7509, 6602, 5171, and 3148 *Lm* ^−2^ *h* ^−1^, correspondingly, with a constant external pressure of 9.6 × 10^−13^ bar calculated from the weight of the liquid. The pure water permeance can be derived by normalizing the flux by the pressure drop, and the obtained pure water permeance values are 7.8 × 10^5^, 6.9 × 10^5^, 5.4 × 10^5^, and 3.3 × 10^5^ *Lm* ^−2^ *h* ^−1^ bar^−1^, respectively (Fig. 7f). From the result, the water flux increases with decreasing cooling rate due to the larger channel size.

Herein we use Darcy’s law to briefly explain how flow rate is increasingly limited when the channel widths of membrane are decreased as shown in Table S2. A volumetric flow *Q* of a fluid of viscosity *μ* pass through a membrane can be expressed by:

4 $${\rm{Q}}=-\frac{kA{\rm{\Delta }}P}{\mu L}$$

where *A* is the cross-section area. *k* means permeability, and *L* is thickness. A transmembrane pressure can be expressed as Δ*P*. By setting the transmembrane pressure to the breakthrough pressure, we can express the maximum flow rate as follows^41^:

5 $${Q}_{max}=\frac{{\rho }_{P}A{\gamma }_{wo}\pi \,\cos \,{\theta }_{adv}{r}^{3}}{4\mu L}$$

where *ρ*_*P*_ is the number pore density, *γ*_*wo*_ is the interfacial tension between water and oil, r is the radius of pore, and *θ*_*adv*_ represents the advancing contact angle of a water droplet on the membrane surface under oil. The high water flux of our samples is contributed mainly by three factors. First, the superhydrophilicity of the plate makes it completely wetted and infiltrated by water rapidly. Secondly, the micro-scale channels created by freeze casting and the nano-sized pores on the diatomite increase the water permeability. Finally, the lamellar structure aligned parallel to the longitudinal direction can reduce the path length for water transportation, letting the water flow down easily. The pure water flux and permeance of diatomite membrane are comparable to the reported work on separation membrane and stainless steel meshes under externally applied pressures^5, 8, 42, 43^. It is ~175 times higher than that of a typical industrial ultrafiltration membrane unit^44^. Diatomite membranes have well-aligned channels along the freezing direction with controllable channel size, resulting in high water flux and permeance.

## Conclusions

In this study, we successfully synthesize a hierarchically-porous membrane by freeze casting technique using diatomites as raw materials. The structural morphology and surface wettability are characterized and discussed. The diatomite membrane is applied to the treatment of oily waste water, and its separation performance, stability, durability, and recyclability are investigated. Hierarchically-porous structures with micro-scale inter-connected channels aligned uni-axially in the longitudinal direction are developed, and sophisticated nano-scale porosities are well-preserved on the walls of frustules. The diatomite membrane shows superhydrophilicity and superoleophilicity in air due to the synergistic effect of surface roughness and hydrophilic nature of diatomite. When immersed in water, the porous structure traps a lot of water within the channels and pores, forming stable oil/water interface in the presence of oil, thus exhibits superoleophobicity to various kind of oils underwater. Such high selectivity towards oil and water induces over 99.6% separation efficiency for free oil/water mixtures, driven solely by gravity. Water permeance of diatomite membrane is measured to be one or two order higher than those synthesized by conventional techniques. The intrusion pressure can reach more than 7.2 kPa of soybean oil due to the stable oil/water interface created by diatomite membrane. High intrusion pressure makes it capable of treating large amount of oily waste water, and has potential for large scale separation. Additionally, the diatomite membrane can sustain severe environments, including strong acid and brine, due to the chemical stability of inorganic silica compared with conventional polymeric membranes. Moreover, the plate displays great anti-oil-fouling property as well as mechanical stability and can be reused for over 30 separation cycles without degradation, demonstrating good durability and recyclability, which can be potentially applied for oil/water separation.

## Methods

### Materials and Reagents

Food-grade diatomite powder (Fossil Shell Flour^®^) was purchased from Perma-Guard Inc., USA. Polyvinyl alcohol (PVA, 98–99% hydrolyzed, average molecular weight (M.W.) 88,000–97,000, Alfa Aesar, USA), polyethylene glycol (PEG, average M.W. 5400–6600, Alfa Aesar, USA), and sodium polyacrylate anionic dispersant (DARVAN^®^ 811, Vanderbilt Minerals, USA) were acquired and used as received. The model oils used in this research were soybean oil/sunflower oil (Uni-President Enterprises Corp., Taiwan), hexane (>99%, Avantor Performance Materials, USA), n-dodecane (95%, Tedia, USA), n-hexadecane (>98%, Tokyo Chemical Industry, Japan), n-octane (99+%, anhydrous, Aldrich, USA), n-undecane (99%, Acros organic, USA), and crude oil (CPC Corp., Taiwan). Sea water was obtained from Nanliao Harbor, Taiwan, and stored at 7 °C. The artificial sea water was synthesized according to ASTM D1141-98^40^.

### Preparation of diatomite scaffold and diatomite membrane

The diatomite scaffolds were synthesized by well-controlled freezing of diatomite slurry which was composed of diatomite powder, organic binder and anionic dispersant. The organic binder was prepared by adding 1 wt% of polyvinyl alcohol and polyethylene glycol into deionized water. The mixture was heated up to approximately 90 °C to dissolve all the ingredients. Followed by mixing 3 wt% of anionic dispersant and 18.5 wt% of diatomite into the organic binder for 15 minutes, the diatomite slurry was obtained. The as-synthesized diatomite slurry was poured into the customized freeze casting system. A copper cold finger was immersed in a nitrogen bath and connected to a ring heater with a proportional-integral-derivative (PID) controller to create a steady and controllable cooling flux. The top of the copper cold finger was inserted into a hollow PTFE tube. The process temperature was decreased from 10 °C to −140 °C with adjustable cooling rates. After the cooling process, the frozen slurry was put into the freeze dryer (FD 4.5/−80, Firstek, Taiwan) at a low temperature and reduced pressure (<−80 °C and 50 mTorr) to sublimate the ice and preserve the porous structure of the green body. After idling for 48 hours, the green body was put into an open-air box furnace (Lindberg/Blue M™ BF51314C, Thermal Product Solutions, USA), and sintered at 1050 °C for 4 hours. For the following measurement of surface wettability and oil/water separating performance, the scaffolds were sectioned into thin diatomite membranes with 2 mm in thickness by a rotating diamond blade.

### Characterization techniques

The surface morphology and topography of samples were characterized by a FE-SEM (SU-8010 Hitachi, Japan), and the phases were identified by an XRD (XRD-6000, Shimadzu Co., Japan). The surface roughness was analysed from the three dimensional topography images by a confocal white light optical microscope (nanofocus *μsurf C*, NanoFocus, Germany). The objective lens is 20X 800 S of optical module with 3.1 mm of working distance, and 800 × 800 *μm* ^2^ of measuring capture field. Considering the image quality, it is not to go for higher magnification because of severe light scattering by such a porous structure. For each membrane, N = 20 roughness profile were measured in order to calculate a mean root-mean-square roughness (R_q_) and standard deviation. The root-mean-square surface roughness (S_q_) was measured from 5 measuring capture field for each scaffold. The roughness parameters and waviness all conform with DIN EN ISO 4287 and DIN EN ISO 4288. The wettability measurement was carried out by a contact angle goniometer (FTA-1000B, First Ten Angstroms, USA). For oil/water contact angles measured in air, a sessile drop method was applied, and liquid volume of a drop was controlled at 5 µL. The static contact angle was measured after the liquid droplet contacted the surface for 5 seconds. For underwater oil wettability measurement, the sample was fixed upside down in a transparent quartz container filled with water. Since the model oils had smaller density than water, an oil droplet was dispensed out from an inverted J-shape needle under the sample, and moved upward slowly until the edge of the oil drop reached the substrate without observable gap. Then the oil drop continued to move up for another small distance, approximately 0.2 mm, to ensure the contact. At least 5 individual measurements were performed on different locations of each sample to obtain an average value.

### Oil/water separation

The hierarchically-structured diatomite membrane was fixed between two glass tubes. Before the separation, the membrane was firstly wetted by water and 20 mL of free oil/water mixture (V_water_/V_oil_ = 1:1) was poured into the upper tube. The only driving force during the whole separation process was gravity. After the separation, the system was idle for approximately 10 minutes, and the filtrate was observed under an optical microscope to check if any oil residue penetrated through the plate. The oil was dyed red by Oil Red O (Acros Organics, USA) and water was dyed blue by Methylene Blue (Acros Organics, USA) for clear observation.

## Electronic supplementary material

Bioinspired Diatomite Membrane with Selective Superwettability for Oil/Water Separation
