# Supplementary material for: Bioinspired Diatomite Membrane with Selective Superwettability for Oil/Water Separation
Source: Sci Rep. 2017 May 3;7:1426. doi: 10.1038/s41598-017-01642-2 (PMC5431206; doi:10.1038/s41598-017-01642-2)
Supplement: Supplementary file 1 — Bioinspired Diatomite Membrane with Selective Superwettability for Oil/Water Separation [file 41598_2017_1642_MOESM1_ESM.doc]

Supporting Information

Bioinspired Diatomite Membrane with Selective Superwettability for Oil/Water Separation

**Yu-Hsiang Lo ^a+^, Ching-Yu Yang ^a+^, Haw-Kai Chang ^a^, Wei-Chen Hung ^a^ and Po-Yu Chen ^a,*^**

Department of Materials Science and Engineering, National Tsing Hua University, Hsinchu 101, Sec. 2, Kuang-Fu Rd., Hsinchu 30013, Taiwan

*Corresponding author: poyuchen@mx.nthu.edu.tw

+ Y.-H. Lo and C.-Y. Yang have equal contribution to the manuscript

**Tables:**

**Table S1.** The underwater contact angles (
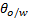
) of membranes synthesized with cooling rate of 5
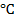
/min, tested by different oils. Each shows stable superoleophobicity.

| Tested oils | Surface tension at 25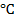  (mN/m) | 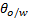 (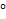) |
| --- | --- | --- |
| Soybean oil | 30.5 | 169.4 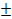 1.3 |
| Hexane | 18.4 | 167.1 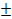 0.4 |
| Hexadecane | 27.5 | 167.0 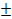 0.9 |
| Dodecane | 25.4 | 168.3 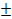 0.7 |
| Light crude oil | -- | 170.2 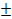 1.7 |
| Heavy crude oil | -- | 171.1 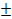 1.3 |

Table S2. Surface tensions of tested oils for superhydrophobicity underoil and the corresponding contact angle of water under oils (
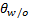
).

| Oils | Surface tension (mN/m) | 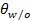 (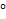) |
| --- | --- | --- |
| Hexane | 18.4 | 168.1 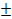 2.3 |
| Octane | 21.6 | 164.2 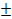 3.1 |
| n-Undecane | 24.7 | 158.5 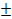 2.1 |
| Hexadecane | 27.5 | 156.2 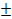 2.4 |
| Soybean oil | 31.2 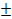 1 | 154.2 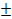 3.1 |
| Sunflower oil | 33.5 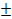 0.5 | 167.5 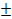 5.1 |
| Sesame oil | 26.5 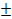 1 | N/A |

**Table S3.** The effect of cooling rates on the microstructural features of scaffolds and water permeance.

| Cooling rate (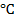/min) | Channel width (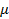m) | Lamellae width (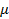m) | Permeance (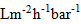) |
| --- | --- | --- | --- |
| 2 | 13.3 ± 5.7 | 14.7 ± 5.7 | 7.8 × 10^5^ |
| 5 | 11.1 ± 3.3 | 13.4 ±3.9 | 6.9 × 10^5^ |
| 10 | 11.7 ± 2.9 | 12.6 ±3.1 | 5.4 × 10^5^ |
| 15 | 8.8 ± 2.6 | 6.2 ± 1.9 | 3.3 × 10^5^ |

**Figures:**


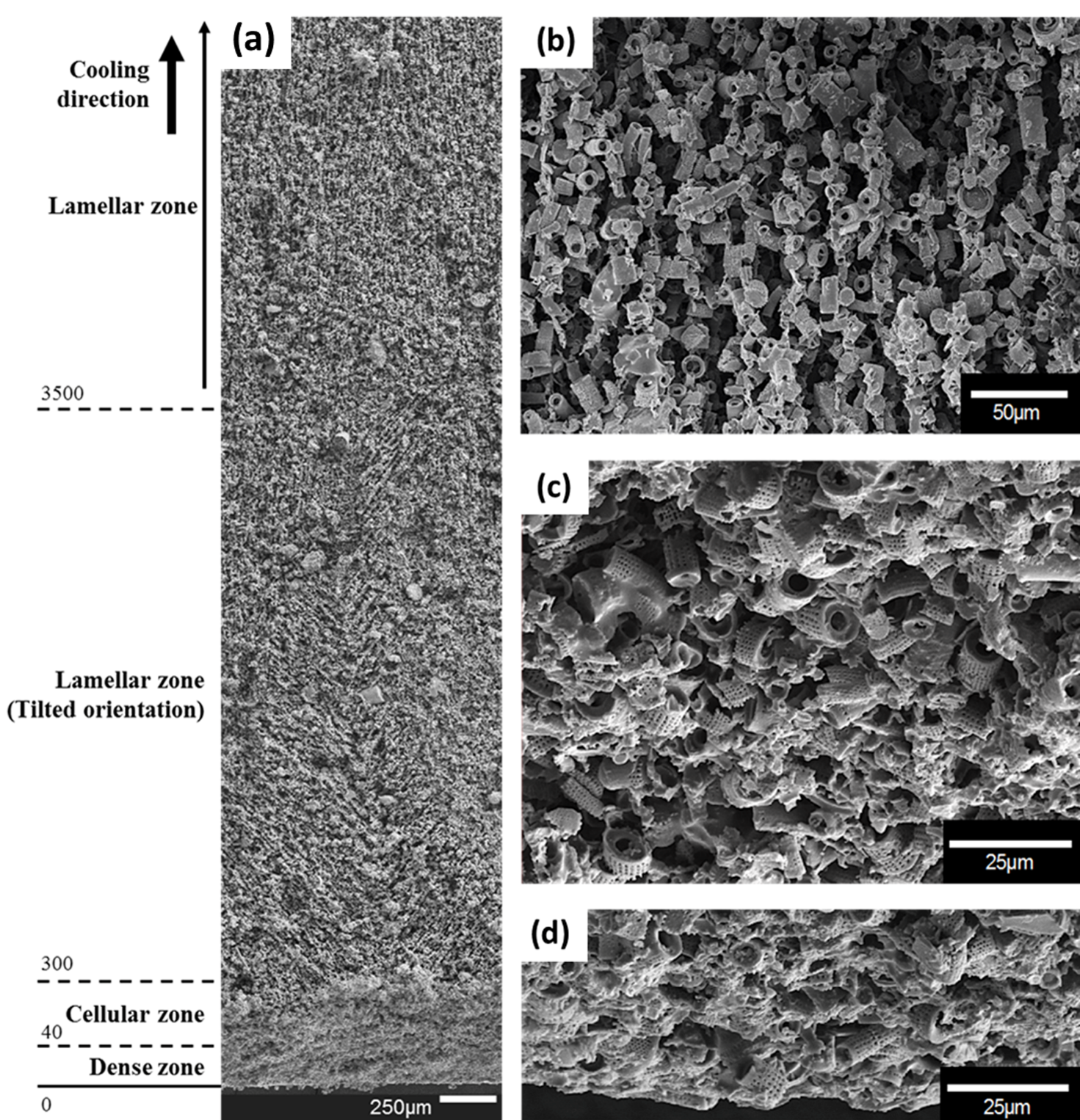


Fig. S 1. (a) SEM image shows the longitudinal section of a synthesized diatomite scaffold. SEM images under higher magnifications show three distinctive regions, (b) lamellar (c) cellular and (d) dense zones.


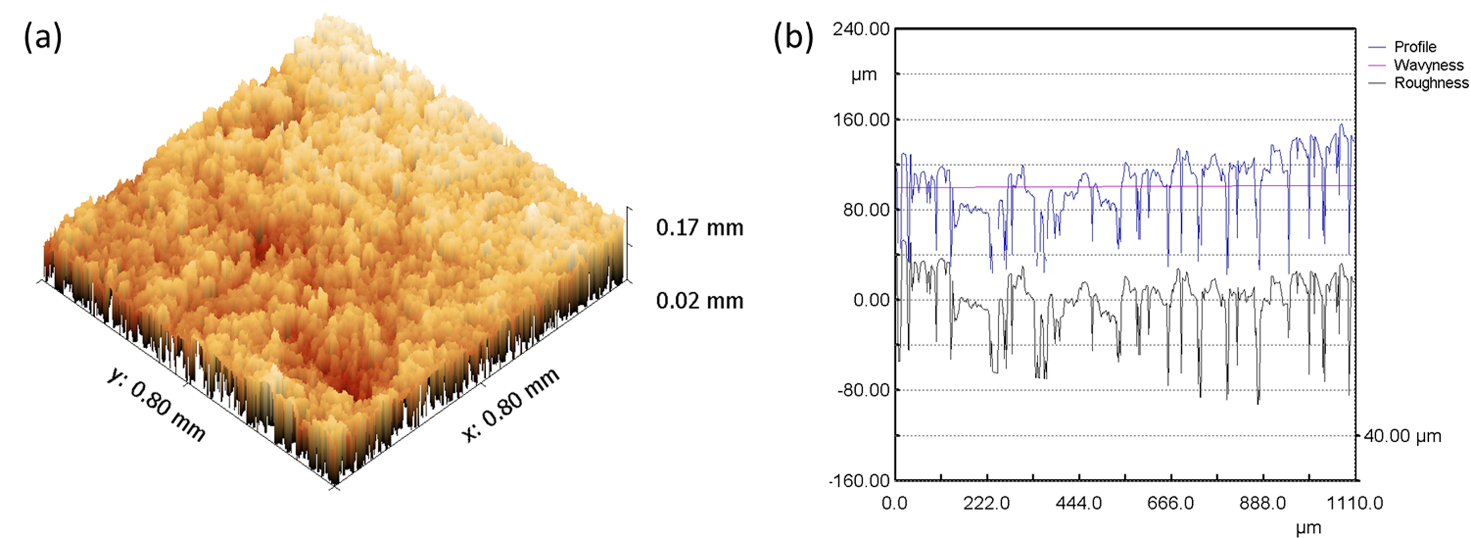


Fig. S 2 (a) A image of topography for diatomite membrane. (b) The corresponding surface profile and the calculated roughness profile.


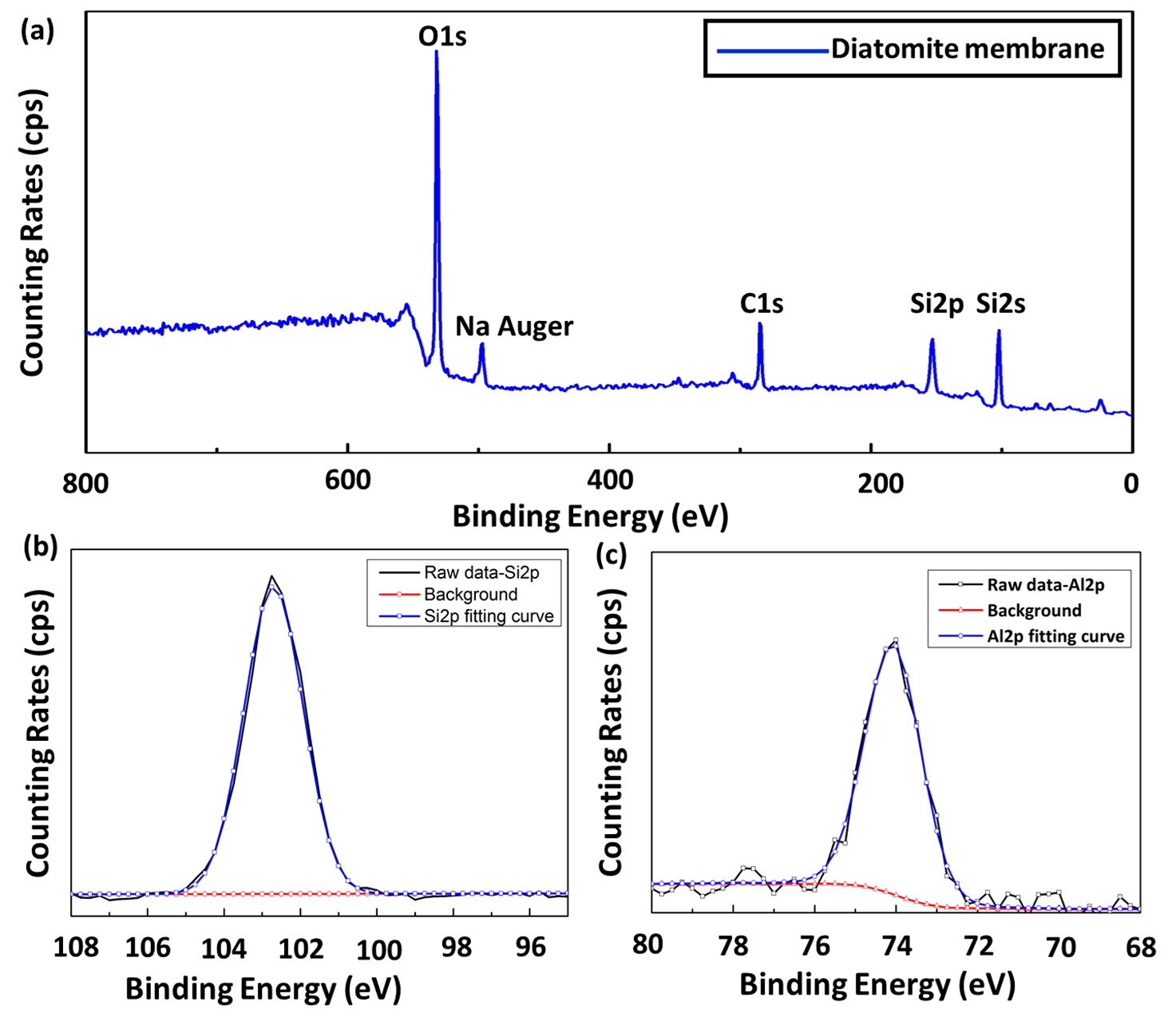


Fig. S 3. (a) Full XPS spectrum of diatomite membrane. XPS spectra of (b) Si2p and (c) Al2p, respectively.


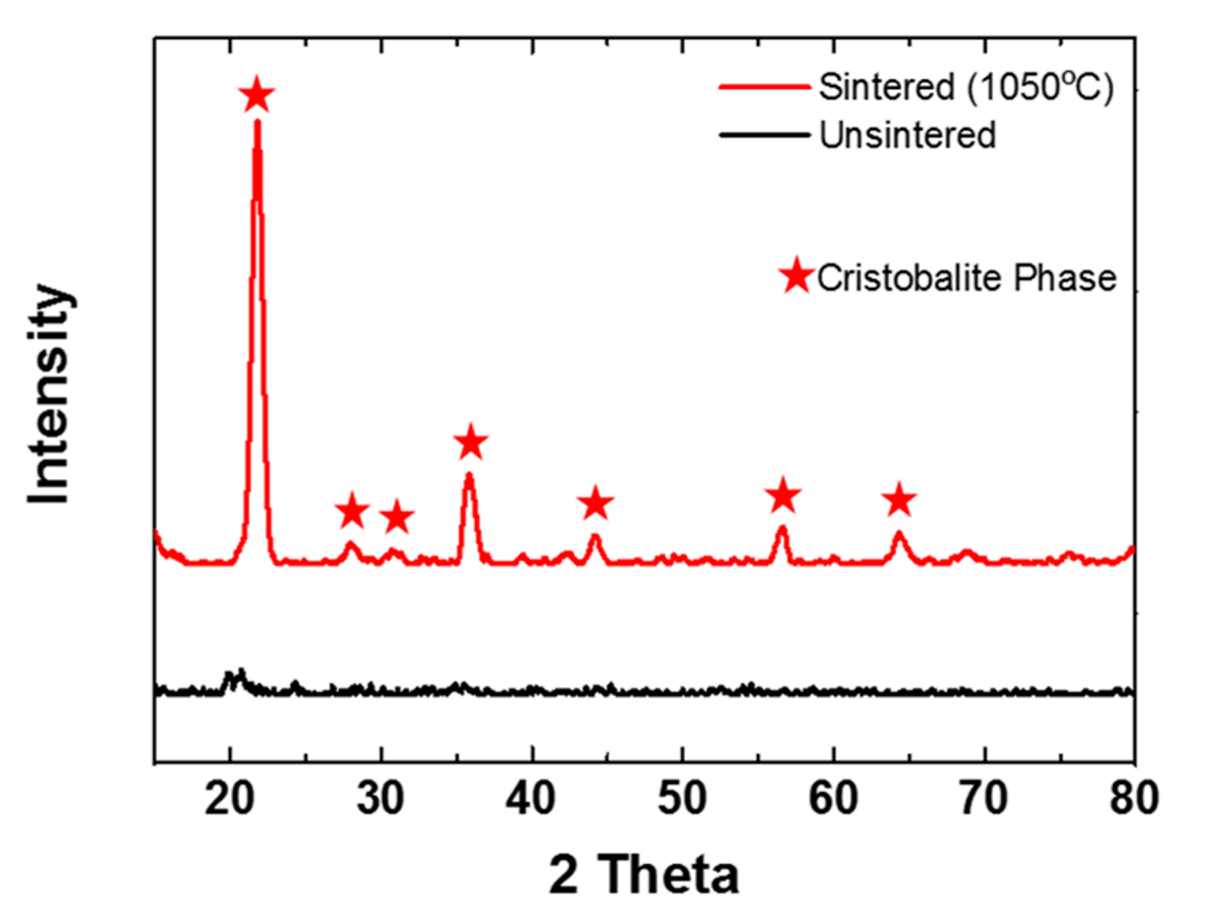


Fig. S 4 XRD spectra of the diatomite powder before and after sintering at 1050
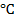
.


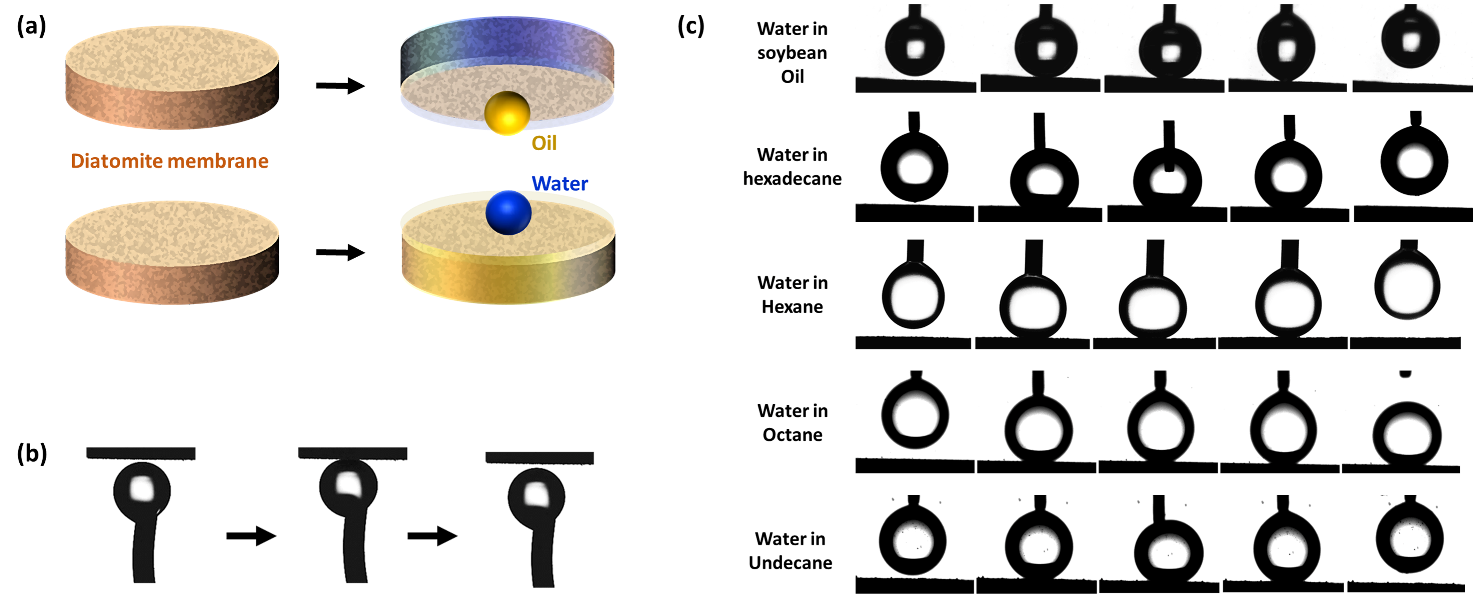


Fig. S 5. (a) Schematic illustrations of oil wetting on a freeze casted, the diatomite membrane with hierarchical micro/nano-structures in water-oil systems. (b) Still images capture from a video showing a soybean oil droplet (model oil for underwater superoleophobicity) approach, compress, and leave the surface. No residue is left on the surface after separation. (c) Still images showing a series of oils are repelled by the oil-prewetted membranes.


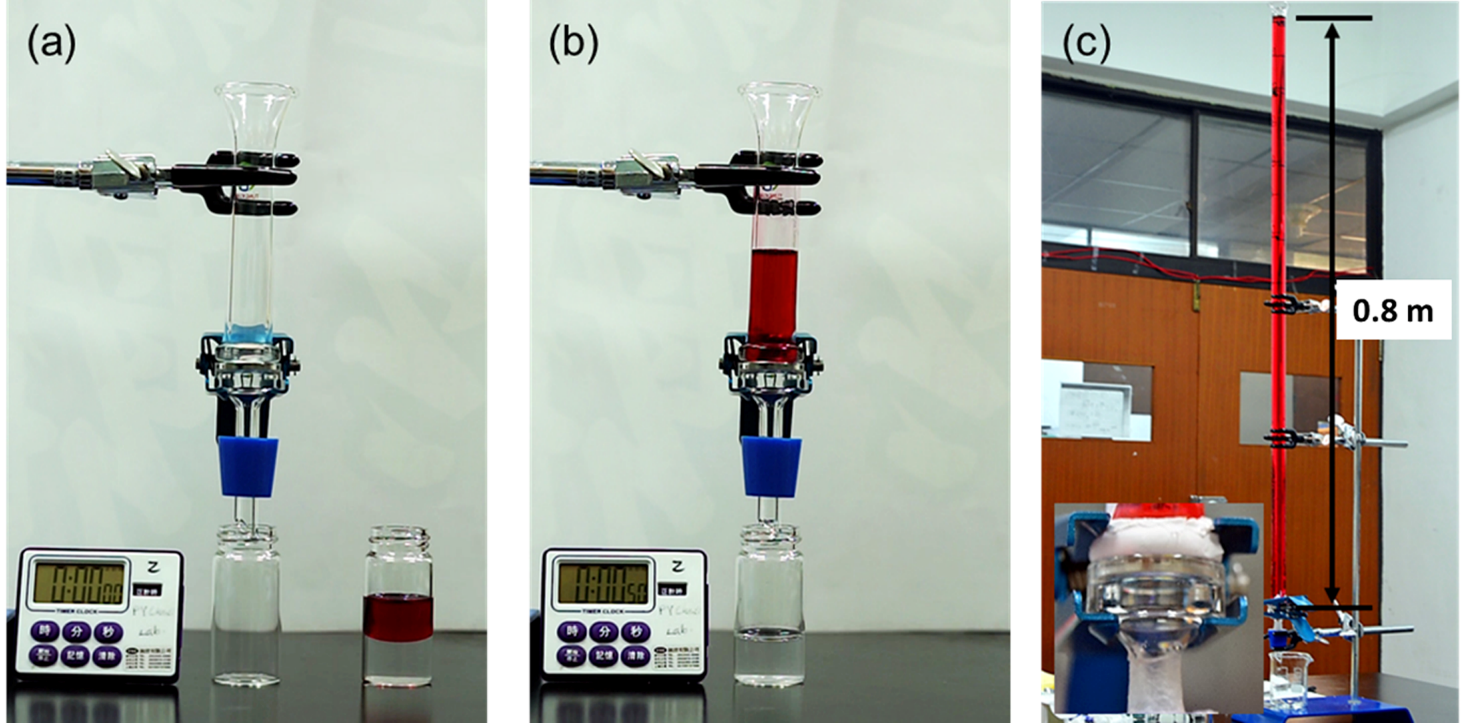


Fig. S 6. (a)-(b) Oil/water separation process. (c) Scaffolds with cooling rate of 5
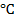
/min can sustain over 0.8 m of soybean oil. Inset shows no visible oil penetrates the material during the experiment.


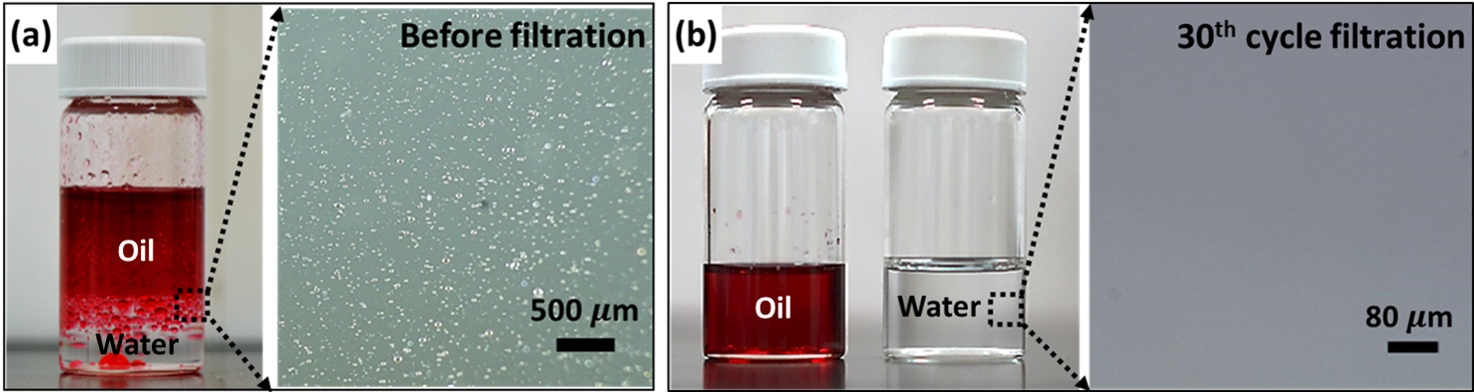


Fig. S 7. The photographs and the corresponding optical microscope (OM) images for prepared free soybean oil/water mixture: (a) before separation; (b) the liquid collected after 30 cycles of separation. The OM image of the filtrate shows no oil residue and satisfactory durability.


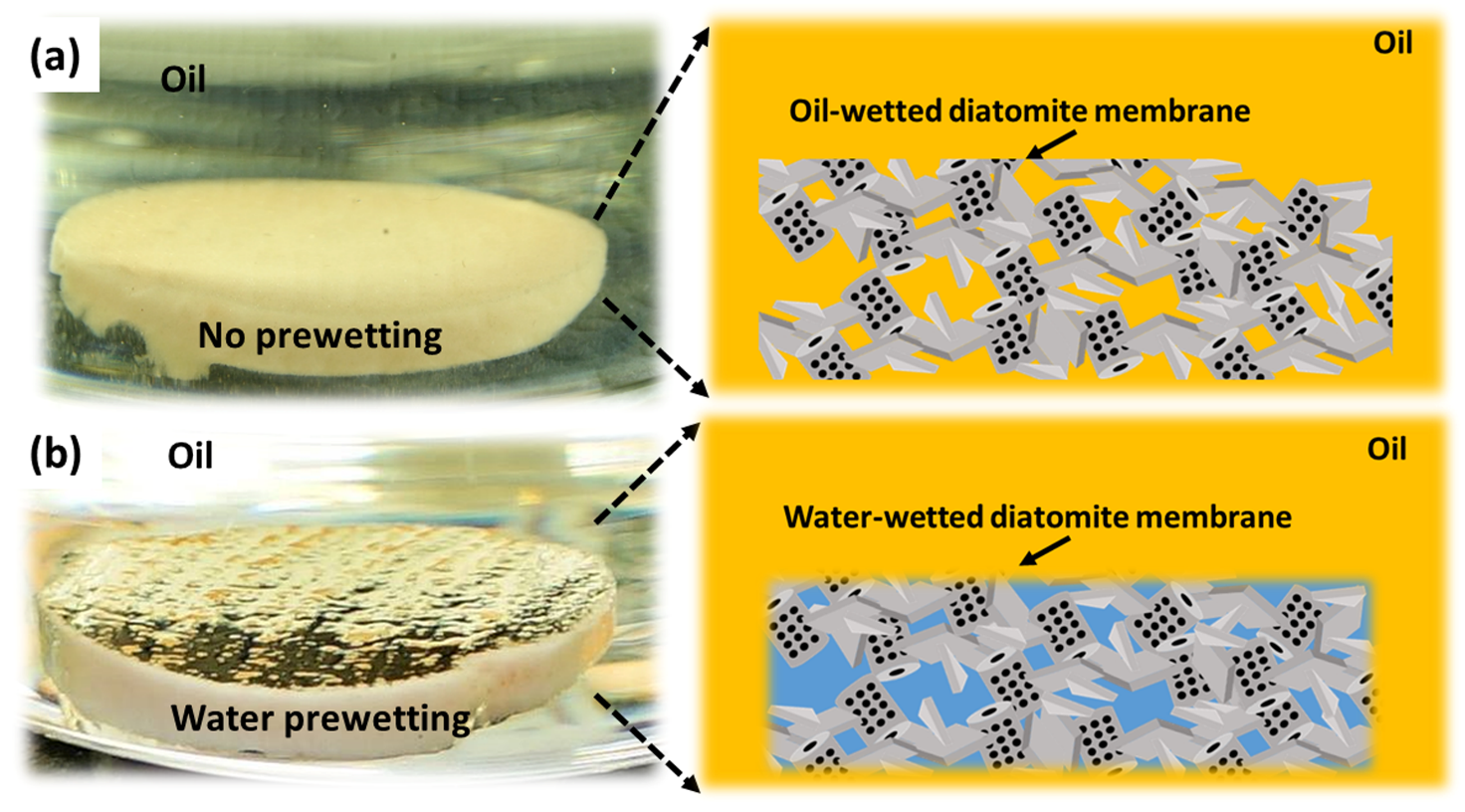


Fig. S 8. The photographs and illustrations of (a) oil-wetted diatomite membrane without prewetting by water and (b) water pre-wetted diatomite membrane in oil bath.
